# Some effects of a chrysin bromide-derivative on GABA-A receptors and on Caenorhabditis elegans

**DOI:** 10.17912/micropub.biology.000083

**Published:** 2019-01-28

**Authors:** José Luis Téllez-Arreola, Alejandro Valdez-Calderón, Simplicio González-Montiel, Ataúlfo Martinez-Torres, Adan Hernandez

**Affiliations:** 1 Departamento de Neurobiología Celular y Molecular, Laboratorio de Neurobiología Molecular y Celular, Instituto de Neurobiología, Universidad Nacional Autónoma de México, Juriquilla, 76230 Santiago de Querétaro, Querétaro, Mexico; 2 Area Académica de Química, Centro de Investigaciones Químicas, Universidad Autónoma del Estado de Hidalgo, km. 14.5 Carretera Pachuca-Tulancingo, Ciudad del Conocimiento, C.P. 42184, Mineral de la Reforma, Hidalgo, Mexico; 3 Universidad Tecnológica de la zona Metropolitana del Valle de México, Blvd. Miguel Hidalgo Y Costilla 5, Los Heroes, 43816 Tizayuca, Hidalgo, México; 4 Department of Biology, University of Utah, Salt Lake City, UT 84112, USA; Howard Hughes Medical Institute

**Figure 1:**
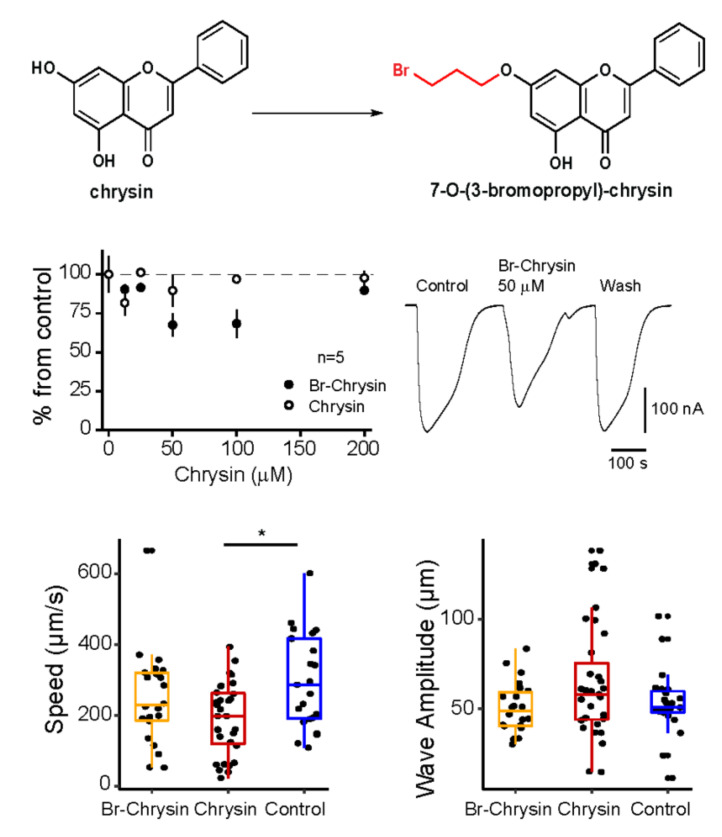
Structure and effects of chrysin and its Br-derivative. A) Structure of chrysin and 7(3-bromopropoxy)-chrysin, B) representative GABA-currents in presence or absence of 7(3-bromopropoxy)-chrysin. Percent of change of the GABA-induced current amplitude in presence of chrysin and chrysin-derivative at different concentrations. C) Speed and wavelength of the nematodes in the presence of the compounds after 3 h of exposure to 100 µM in each condition.

## Description

Infections by helminths are one of the major health problems in developing countries. Fast adaptation of parasitic organisms has conferred resistance to drug therapies used for decades (Burns et al., 2015), causing a threat to control worm parasites and generating an urgent need for new emergent molecules with therapeutic potential, in particular for nematodes. The nematode *Caenorhabditis elegans* is phylogenetically-related to parasitic species of helminths in which high throughput assays are challenging; thus, this species is an excellent model to study and evaluate novel anthelmintic drugs (Gilleard, 2004; Yoon et al., 2006). One interesting molecular target for drug development are the GABA receptors of parasitic helminths which are evolutionarily preserved in invertebrates and vertebrates and are present in *C. elegans* (Castillo et al., 1967; del Castillo et al., 1964; Jorgensen, 2005). GABA receptors from *C. elegans* are ionotropic channels that belong to the Cys-loop superfamily of ligand gated ion channels widely spread in multiple species, including humans (Olsen and Sieghart, 2008).

Flavonoids are natural products of fruits and vegetables commonly present in the human diet. They exhibit a wide range of biological activities such as antibacterial, anti-inflammatory, antiallergic, antithrombotic, vasodilatory and anticarcinogenic. Furthermore, flavonoids are modulators of human ionotropic GABA receptors (Goutman et al., 2003). In recent years, chrysin, a 5,7-dihydroxyflavone produced in plants that elicits hyperalgesia via GABA-A receptors (Kui Zhai et al., 2008), has been chemically modified to test its anthelmintic activity (Rendon-Nava et al., 2017). Here we modified chrysin at the 7-OH by introducing alkyl bromide groups to generate its derivative: 7-O-(3-bromopropyl)-chrysin. To evaluate its activity, we employed two strategies:

1.We injected the mRNA of human GABA-A rho1 receptor in *Xenopus laevis* oocytes (Martinez-Torres et al., 1998) and after 2-4 days its effect was tested on GABA-currents generated by perfusing GABA (3 µM), these basal currents were used as control. GABA-currents were not significantly modified in presence of chrysin. In contrast, 50 µM of 7-O-(3-bromopropyl)-chrysin reduced the amplitude of GABA-currents when perfused onto the bath (reduction to 67.5 ± 6.8 % from control) and 100 µM (reduction to 68.4 ± 8.7 % from control): neither chrysin or 7-O-(3-bromopropyl)-chrysin induced any evident current on the oocytes, not induced evident changes in these cells.

2. We tested whether chrysin and 7-O-(3-bromopropyl)-chrysin elicits changes on locomotion on *C. elegans*. The speed of displacement of the worm and the wave amplitude of its shape in free-movement were evaluated after 3 h of drug exposure. Results showed that chrysin at 100 uM reduced the speed movement without changes on the wave amplitude of the nematode. 7-O-(3-bromopropyl)-chrysin did not elicit significant difference compared to the control. Exposure to the compounds for more than 24 h did not show further effects.

The insertion of an aliphatic bromide chain in the OH of chrysin’s position 7 (Figure 1A) potentially enhance the lipophilic capacity of the compound due to the presence of the methylene group. We expected these modifications to enhance cell permeability and that the presence of a halide to promote ionic interactions. However, the results showed that 7-O-(3-bromopropyl)-chrysin partially blocks GABA-A rho1 receptors expressed in frog oocytes (Figure 1B). In *C. elegans*, reduction of speed movement could be due to the inhibition of native GABA-receptors normally expressed in muscle and neurons, thus affecting locomotion. We expected that the amplitude of the shape was modified as a result of the effects on muscle contraction, but we did not find evidence of such effect.

In conclusion, we report that the chrysin derivative 7-O-(3-bromopropyl)-chrysin negatively modulates human ionotropic GABA-A rho1 receptors expressed in frog oocytes. Chrysin reduces significantly the speed movement of *C. elegans*, however the 7-O-(3-bromopropyl)-chrysin does not have effect, in summary chrysin could be potentially used as template to generate new drugs for anthelmintic properties or synaptic modulators.

## Methods

Br-derivatives of chrysin were prepared as previously described (Babu et al., 2006, Li et al., 2009, Valdez et al., 2016). A mixture of chrysin and anhydrous K_2_CO_3_ equimolar in anhydrous acetone was refluxed until the solution became clear. Then, the crude reaction was allowed to cool down, obtaining a yellow solid which was filtered, dried and purified by column chromatography (CC) on silica. The role of chrysin and 7-O-(3-bromopropyl)-chrysin on GABA-induced currents was evaluated in frog *X. laevis* by two-electrode voltage clamp technique measuring the peak amplitude in presence of the drug in the bath solution. Ovarian lobes were removed from anesthetized frogs, the follicular layer was removed manually and with collagenase (0.3 ug/ul), before of the oocyte microinjection. Oocytes were injected with 50 nL of human GABA-A rho1 mRNA (1mg/ml) and expression assessed after 2-4 days. Drugs were prepared in DMSO (1000X times concentrated) and diluted to 100 µM concentration in Ringer’s solution.

The worms were synchronized and selected in young adult stages. They were placed on plates containing each of the compounds diluted on 1% DMSO to be evaluated at a concentration of 100 µM (7-O-(3-bromopropyl)-chrysin, chrysin and M9+1% DMSO as a control) for a period of 3 h. Subsequently, video recordings of movement for each group were made at intervals of 1 min every 1 h. Videos were taken at 10 fps at 1024 X 1024 pixels with a high-speed camera PCO edge 4.2. Videos were analyzed with Wormlab 3.1 software.

Statistical analysis. Three independent replicates were made from each experimental group. For the analysis of the data, the Shapiro Wilk and Bartlett test were used. The data are represented by the medians of the groups. A one-way Kruskal-Wallis test was applied for the analysis of variance between groups. The statistical value used is p <0.05

## Reagents

The strain used in this study was N2 as a wild-type.

All chemicals commercially available were used without further purification. Chrysin (C80105) and 1,3 dibromo propane (125903) were acquired of Sigma-Aldrich.
